# Extravasation Injuries in Adults

**DOI:** 10.1155/2013/856541

**Published:** 2013-05-08

**Authors:** S. Al-Benna, C. O'Boyle, J. Holley

**Affiliations:** Department of Burns and Plastic Surgery, Nottingham City Hospital, Hucknall Road, Nottingham NG5 1PB, UK

## Abstract

Insertion of an intravascular catheter is one of the most common invasive procedures in hospitals worldwide. These intravascular lines are crucial in resuscitation, allow vital medication to be administered, and can be used to monitor the patients' real-time vital parameters. There is, however, growing recognition of potential risks to life and limb associated with their use. Medical literature is now replete with isolated case reports of complications succinctly described by Garden and Laussen (2004) as “An unending supply of “unusual” complications from central venous catheters.” This paper reviews complications of venous and arterial catheters and discusses treatment approaches and methods to prevent complications, based on current evidence and endeavours to provide information and guidance that will enable practitioners to prevent, recognise, and successfully treat extravasation injuries in adults.

## 1. Definition

Extravasation injury is defined as the damage caused by the efflux of solutions from a vessel into surrounding tissue spaces during intravenous infusion. The damage can extend to involve nerves, tendons, and joints and can continue for months after the initial insult. If treatment is delayed, surgical debridement, skin grafting, and even amputation may be the unfortunate consequences of such an injury [1].

## 2. Incidence

Extravasation is not as rare as many people think, and it may occur even in the most closely monitored situations. A study which investigated extravasation over a five-week period in a UK hospital established an incidence of 39% in adults, almost double that of previously published reports [2]. Two percent of the Medical Defence Union cases involving anaesthetic-related events between 1970–1982 (excluding deaths) were due to extravasation injuries [3], and for those patients who received a course of cytotoxic injections 5% experienced extravasations [4]. Despite this, extravasation injuries remain uncommon, with an estimated incidence published in the literature of between 0.1% and 6% in patients receiving chemotherapy [1]. The published rate is likely an underestimation, however, as many cases of extravasation go unreported.

## 3. Aetiology

Sites most often implicated in extravasation injuries include the dorsum of the hand and foot [5], ankle, antecubital fossa [6], and near joints or joint spaces [7] where there is little soft tissue protection for underlying structures [8]. Limbs with local vascular problems such as lymphoedema may have reduced venous flow causing pooling and potential leakage of infusates around the site of cannulation [5, 9]. Peripheral rather than central venous administration of antineoplastic agents is more likely to be associated with frequent cannulation which is a risk factor for extravasation, and this should be avoided [5, 7].

More extravasations occur at night and often go unnoticed [10]; however, data from the National Extravasation Information Service green card reporting database shows that 44% of extravasations occur between the hours of 2 pm and 10 pm, 10% occur between 10 pm and 6 am, and 38% occur between 6 am and 2 pm [11]. Inexperienced personnel may pose a higher risk, particularly during cytotoxic administration.

There are various patient factors that contribute to the aetiology of extravasation injuries. Veins of people receiving chemotherapy for cancer are often fragile, mobile, and difficult to cannulate [12, 13]. Patients who receive chemotherapy at the same site as radiotherapy may experience a reactivation of skin toxicity known as ‘‘recall” phenomenon [9, 14–17], and patients who have had an extravasation and receive further chemotherapy in a different site may experience an exacerbation of tissue damage in the original site. Patients who have undergone radical mastectomy, axillary surgery, or lymph node dissection may have impaired circulation in a particular limb which reduces venous flow and may allow intravenous solutions to pool and leak out [18].

Diabetic patients with peripheral neuropathy may not experience the pain of an infusate leaking into the subcutaneous tissues [2, 13, 19, 20], and those who suffer from superior vena cava syndrome have persistently elevated venous pressure which, again, may predispose to leakage at the intravenous site [5, 13]. Another more obvious risk is the one posed by intravenous drug users where vessels are often thrombosed and the number of accessible veins is limited, but those who require repeated infusions for their medical condition may experience the same difficulties [13, 21–23].

Patients with other diseases can have increased risk of extravasation injuries because of the same mechanism. This reduction in venous flow and risk of leakage at the intravenous site have been observed in patients with peripheral vascular disease [13, 18, 20, 24, 25] and in those with Raynaud's phenomenon where arterial spasm may compromise the peripheral circulation [2, 19, 20]. The treatment of these diseases may also represent an increased risk of extravasation injury as shown in Table 1.

## 4. Pathogenesis

Complications of peripheral venous catheters include thrombophlebitis, infection, and extravasation injuries. Thrombophlebitis from peripheral venous catheters is a relatively uncommon complication, probably because the average dwell time for such devices is relatively short. Thrombophlebitis can be relatively benign, with redness and tenderness over the course of the vein which resolves after discontinuation of the infusion, or it can present as a more serious suppurative complication, where bacteraemia and metastatic foci of infection occur [26].

Soft tissue damage following extravasation may be due to a number of factors related to the physicochemical properties of the drug or infusate. The following agents have been known to cause extravasation injuries, but the lists are by no means definitive. The most important input that a pharmacy can have is by considering the drugs themselves and by characterising their extravasation risk. It is now well documented that a number of physicochemical factors influence, and usually increase, the extravasation risk of individual drugs as follows:

- ability to bind directly to DNA,
- ability to kill replicating cells,
- ability to cause tissue or vascular dilatation,
- pH outside the range 5.5–8.5,
- osmolarity greater than plasma (>290 mosmol/L),
- formulation compounds such as alcohol, polyethylene glycol.

With extravasation injuries, the degree of cellular injury is determined by the volume of the infiltrating solution and physicochemical characteristics, such as pH, osmolarity, and degree of dissociability (pKa). Infiltration of vasopressors such as dopamine and adrenaline produces intense local vasoconstriction and tissue ischaemia [10, 27], and in contrast, vasodilators may exacerbate the effects of extravasation by increasing local blood flow and enlarging the area of injury. Parenteral alimentation fluids, antibiotics, calcium, potassium, and sodium bicarbonate solutions also have the potential to cause severe tissue necrosis [10, 28].

Formulation-related parameters include the concentration and volume of the solutions to be administered. Unfortunately, these two parameters are contradictory to each other in so far as the smaller the volume, the less the likelihood of extravasation, but the higher the concentration, the greater the potential for damage should an extravasation occur. As the most common way to decrease the volume is to increase the concentration, juggling these two factors becomes more of an art than a science.

Chemotherapeutic agents have obvious deleterious effects when extravasation occurs and can lead to more severe injury. These drugs can be classified as irritants or vesicants, depending on the potential for localised toxicity and tissue damage. Many chemotherapeutic agents may overlap the definitions of irritants or vesicants and have the capacity to act as either.  Table 2 demonstrates this classification.

Irritants are defined as agents that produce local inflammation, pain, tightness, or phlebitis either at the site of injection or along the vein. Irritants may induce local sclerosis or hyperpigmentation but do not induce tissue necrosis. The symptoms of the local reaction after extravasation are typically self limiting, most commonly without long-term sequelae [29].

Many antineoplastic (Cellular Toxic) agents as

- doxorubicin,
- daunorubicin,
- vincristine,
- vinblastine,
- mitomycin,
- paclitaxel,
- azathioprine,
- acyclovir

are vesicant (i.e., produce blisters) [7, 9], and as well as causing immediate injury may also bind to tissue DNA [30] so that the drug is continually released from dying to healthy cells, resulting in a slow increase in ulcer size over time. Doxorubicin, for example, has been shown to remain in tissue for 5 months after extravasation [31] which means that the injury can present late with extensive tissue destruction [32]. Ulcers caused by these highly vesicant agents usually do not heal and often require plastic surgery and skin grafting [15].

The full effect of the extravasation injury is not usually immediately apparent but may evolve over days or weeks. Early local symptoms of a vesicant extravasation resemble those of an irritant extravasation: local pain, erythema, burning, pruritus, or swelling [33, 34]. Over the course of the reaction, however, as tissue necrosis evolves and becomes clinically apparent, progressive erythema, discolouration, blistering, or desquamation may develop. The severity of the local reaction may vary both upon the agent extravasated and upon the total dose of extravasated material.

The pathogenesis of the severe tissue damage that vesicant chemotherapeutic agents cause is not fully understood. Agents that bind to DNA induce more damage than non-DNA-binding drugs [34, 35]. These agents are taken up by the surrounding cells, causing progressive and prolonged local damage [35]. This has particularly been suggested to be the case for the severe tissue damage seen with doxorubicin extravasation [28]. Additionally, the significant free-radical formation of vesicant agents is suggested as a potential mechanism of the severe necrotic effect [35].

In order for the extravasated compound to do damage, it must move out from the initial site of extravasation [20]. The fact that this movement occurs is evident when we consider that the resultant area of damage is often considerably larger than the initial physical appearance at the time of extravasation.

An understanding of drug or infusate cellular transport process may allow us to better predict the spectrum of damage that may be expected. Some forms of transport mechanism may directly cause cell death because of the rate at which they affect the local cellular environment. Osmotic pressure is such a factor and this is directly related to the osmolality of the administered drug. Osmotic pressure can cause cell death and hence tissue necrosis by cell implosion from hypertonic solutions, or cell explosion from hypotonic solutions; however, the former of these cellular fates is by far the most common. Some substances have the potential to cause tissue damage by having an osmolality greater than that of serum (281–289 mosmol/L) [36].

Hyperosmolar substances such as hypertonic glucose solutions or X-ray contrast media draw fluid from cells resulting in cell death by dehydration, whereas calcium and potassium salts cause cell death by fluid overload. Hypertonic solutions which contain ions and are also acidic are particularly damaging to tissues because they are capable of killing cells by precipitating cell proteins [36]. Calcium chloride, for example, has caused full-thickness skin necrosis, and hypertonic saline is the most common sclerosant associated with necrosis. Parenteral nutrition extravasation is reported more often in children [18] and can cause skin sloughs [10] and limb contractures particularly in premature infants.

The pH of a substance outside of the physiological range may have an adverse effect on tissue [2, 18, 37, 38]. Thiopentone and phenytoin, for example, are highly alkaline and have caused severe injuries including amputations [39]. Other agents are shown as follows:

acidic agents include

- etomidate [pH 3.4],
- amphotericin [pH 5-6],

while alkaline agents include

- thiopentone [pH 10.5],
- methohexitone [pH 11.5–12.2],
- phenytoin [pH 12].

## 5. Presentation

Extravasation of intravenous fluids is marked initially by pain and swelling, which then progresses to blanching, blistering, and discolouration of the skin. Pain is the most useful symptom to alert the administrator to the possibility of a complication.

Induration, erythema, venous discolouration, or swelling may be observed at the site, but it is worth noting that discolouration alone may not indicate extravasation as doxorubicin, epirubicin, and mitozantrone have all been reported to produce this effect when administered intravenously.

Persistent induration often progresses to a dry black eschar in 1 or 2 weeks, which then usually sloughs to reveal an ulcer. Objective staging of extravasations is useful for quality improvement purposes and for deciding the degree of intervention required [40].

## 6. Recognition

A summary of the signs and symptoms above is presented as follows.

Recognition of an extravasation is through

- pain,
- erythema,
- swelling,
- tenderness,
- local blistering (indicative of at least a partial-thickness skin injury),
- mottling/darkening of skin,
- firm Induration,
- ulceration (usually not evident until 1-2 weeks after injury),
- no capillary filling (a white appearance with nonblanching skin indicating full-thickness skin damage).

Note that not all of the above symptoms may be present.

As well as these signs demonstrated from clinical examination, an awareness of peripheral factors, such as attached pumps and monitoring equipment, may highlight a problem earlier.

A reduced rate of flow may be observed when using an infusion pump, and therefore, close observation is necessary. Increased resistance to the administration, once possible changes in the position of the body for example bending of wrist or elbow, cannula support, or bandaging, have been excluded as possible causes, indicates a displaced cannula and the possibility of extravasation. Once the alternative diagnoses have been considered and excluded and one or more of the symptoms are present, the practitioner should proceed on the basis of a diagnosis of extravasation.

A lack of blood return from the cannula is commonly quoted as a sign that extravasation has occurred. It is however, the most misleading of all signs and has been implicated in a number of serious incidents. If there has been extravasation injury and the cannula has become displaced, the act of trying to draw back test for blood return can move the cannula back into the vein while a hole remains in the vein wall in the proximity of the cannula tip. If administration recommences, a larger and more significant extravasation injury then ensues. Alternatively, the bevel of the needle can puncture the vein wall during venepuncture, allowing drug to escape into the tissue whilst the lumen of the needle may still remain in the blood vessel and allow adequate blood return.

## 7. Management

Treatment is determined by the stage of extravasation, the nature of the infiltrating solution, and the availability of specific antidotes. In all cases of infiltration, the intravenous infusion should be stopped promptly, and any constricting bands or tapes should be removed. Treatment protocols for severe extravasations vary from conservative to aggressive management of the acute injury [28, 39, 41, 42], with additional variations in wound management [10, 43, 44].

**Figure 1:**
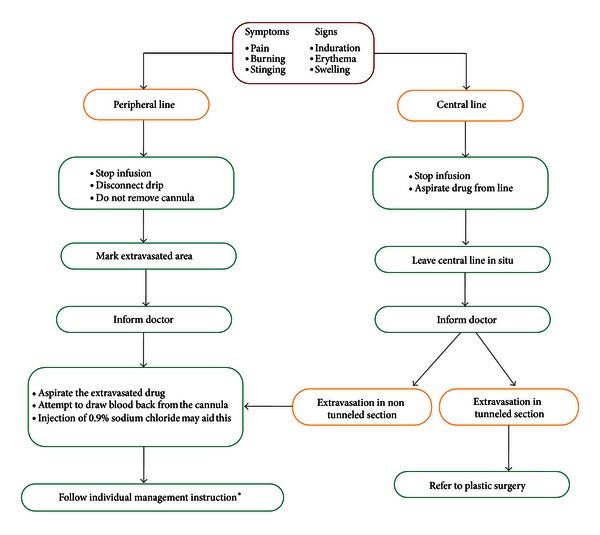
Proposed treatment algorithm. *Individual management instructions. (I) Aspirate extravasation injuries and inject steroid hydrocortisone subcutaneously to the affected area and IV if large-scale inflammation, flare, or fracturing along the vein has occurred. (II) Treatment is then characterised as either (A) spread and dilute (1) using normal saline or hyaluronidase, (2) keep limb warm, (3) use continuous compression and elevation of the limb or (B) localise and neutralise (1) use antidote if available, (2) use intermittent cold compression.

There is no standard treatment for the acute phase of this extravasation injury. However, once it is detected, emergency management must be taken immediately. The infusion should be stopped and the intravenous cannula should be aspirated. Any collection or palpable effusion in the subcutaneous tissues should be drained and the limb should be immobilised and elevated above the heart level. Many authors prefer the conservative treatment until lesions evolve for at least 1 week [13, 34, 45, 46]. On the other hand, with full-thickness skin necrosis, ulcer, or persistent pain, many surgeons suggest early aggressive debridement because the chronicity and the nature of the wound can cause patients to suffer delayed treatment of primary disease (i.e., carcinoma) and morphofunctional damage [13, 45, 46]. In these situations, surgical intervention with radical debridement and wound coverage would be required [39]. A proposed algorithm for approaching the treatment of extravasation injuries is shown in Figure 1.

Treatment of a vesicant extravasation includes immediate cessation of infusion, aspiration of as much extravasated drug as possible through the still-intact catheter, and attempts for the aspiration of the extravasated agent in the surrounding tissue. This aspiration may help to limit the extent of tissue damage. Application of cold packs provides symptomatic pain relief. Hot packs increase local vasodilation, diluting the extravasated drug. Cold packs should not be administered in the event of extravasation of vinca alkaloids as increased tissue ulceration has been demonstrated in animal models with the use of cold packs [47].

The local application of antidotes to different chemotherapeutic agents is based on very limited data. Sodium thiosulfate is recommended as an effective antidote for mechlorethamine cisplatin. Hyaluronidase has been recommended for extravasation of vinca alkaloids [9]. The mechanism of action in prevention of tissue damage is not fully understood and has not been extensively studied. Hyaluronidase has been suggested to act via temporary breakdown of hyaluronic acid, which holds together tissue planes, and subsequent facilitation of drug dispersement and dilution [48].

Topical application of dimethylsulfoxide (DMSO) has been proposed to help prevent significant tissue necrosis in animal and human models. The pathophysiology of the interaction is not known, although free-radical scavenging and facilitation of elimination of drug from local tissues are postulated pathways of efficacy [49]. Procedures such as liposuction or saline flushout have been proposed through a single-institution series but have not been met with widespread usage [50].

Dexrazoxane, employed for protection of anthracycline-induced cardiotoxicity, has been evaluated in animal models and demonstrated to be protective against local tissue damage and ulceration in anthracycline extravasation [51]. Potent free-radical scavenging effects are suggested as the mechanism of protection from tissue damage.

The indications for surgery in an extravasation injury patient include full-thickness skin necrosis, chronic ulcer, and persistent pain [13, 45]. When the patient has fulfilled the indication for surgery, a surgical treatment is necessary as early as possible to decrease the morbidity, suffering, and delayed treatment of primary disease of the patient. It is imperative that complete or radical excision of all necrotic tissues must be performed until the bleeding is observed and only healthy tissue is left for wound coverage. Some authors use the intraoperative fluorescent dye injection to detect the doxorubicin HCl in the tissue to ensure complete excision [52]. Immediate or delayed surgical reconstruction could then be successfully performed [13].

Although case reports of local interventions including glycerine, chlorhexidine, and dimethylsulfoxide (DMSO) have been published for the treatment of docetaxel extravasation, it is not clear whether the application of an antidote for irritant extravasation is more effective than local palliative measures [15].

## 8. Prevention

Measures to prevent extravasation include careful insertion of peripheral venous cannulae, flushing with sterile saline to ensure patency, and suitable dressing to prevent movement, without obscuring possible swelling or erythema. Regular inspection of the site and regulated delivery of intravenous fluids from continuous infusion pumps (usually limited to an hour at a time) may prevent the inadvertent infiltration of a large amount of fluid before detection, but it is helpful to remember that although occlusion alarms on infusion pumps may be set to the lowest limit possible, increased pressure is not always registered [53].

Hyperosmolar fluids, acidic or alkaline solutions, or infusates with irritant or vesicant properties should be given through central venous lines, if possible, or should be diluted or neutralised appropriately. The addition of heparin either to flush solutions or to continuous infusions has not been shown to prolong peripheral catheter patency or to reduce the incidence of infiltration or extravasations conclusively and is not recommended [54].

The site for cannulation must be chosen appropriately to reduce risk of extravasation. This must be in an area where the device can be introduced easily and fastened securely, where it is always in view for regular inspection. Taking these factors into account, the most appropriate site is considered to be the forearm. However, it has to be accepted that this is not always going to be an available area. The vessels in the dorsum of the hand are probably the next most appropriate location to consider. As a general rule joints and creases should be avoided as these often represent a ‘‘small” anatomical space, with nerves and tendons present.

For slow infusion of high-risk drugs, a central line or peripherally inserted central catheter (PICC) line should be used, but if administration through a peripheral cannula is necessary, it is best to administer cytotoxics through a recently sited cannula after ensuring its patency with a saline flush. When administering vesicants by slow intravenous injection, a push into the side-arm port of a fast-running intravenous infusion of compatible solution is recommended. If administering more than one infusion sequentially, the most vesicant drug should be administered first. A frequent assessment of the peripheral site is required, watching for signs of redness or swelling.

If there are any doubts concerning the patency of an intravascular catheter, the infusion must be stopped pending investigation. It is recommended to re-site the cannula if there is any uncertainty about its patency.

Some investigators suggest delaying the administration of antiemetics until after vesicant administration as the sedative and anti-inflammatory effects of antiemetics often mask the early warning signs of extravasation and may impede the patient's ability to report any sensation at the infusion site. It is important to never hurry and to administer drugs slowly to allow the drug to be diluted by the carrier solution while careful assessment of the IV site is undertaken. Documentation of the rate of administration, location and condition of site, verification of patency, and patient's responses, is advised when giving any drugs with the potential to extravasate.

The elimination of human error can be considered to be impossible but systems can be put in place to decrease potential risks and to avoid a ‘‘failure to rescue” scenario. Systems that can be used to minimise this risk include the use of good training and educational policy, not only as stand-alone courses, but importantly, on a continuing educational basis.

## 9. Prognosis

Local necrosis may heal with conservative management, leaving minimal long-term sequelae, or may progress to significant eschar formation and tissue ulceration that ultimately requires surgical debridement and further intervention, with long-term morbidity for the patient.

Ulceration after vesicant extravasation is typically marked by delayed healing. Morbidity may consist of cosmetic defects, chronic pain, or loss of function secondary to contractures or neuropathy, even in the absence of ulceration of skin [48]. Published patient series have estimated that only approximately one third of vesicant extravasations will progress to tissue ulceration [50]. Repeated infusion of the offending agent, even in another limb, may induce a recall reaction at the site of extravasation [14]. One case of squamous cell carcinoma of the skin was documented at the site of a doxorubicin extravasation 10 years previously [50].

When extravasation occurs, there is no certain way of predicting the pattern of damage that will ensue. Heckler [55] proposed clinical staging based on 1 to 4 clinical stages of the extravasation injury. In stages 1 and 2, no signs of skin damage and loss are observed, whereas in stages 3 and 4, the soft tissue damage is more extensive and may include skin and underlying tissue necrosis.

Although there is little direct literature on the effect of time from occurrence to either treatment or extent of maximum injury, all authors make the generalised statement that the sooner an extravasation injury is treated, the better the outcome, and the smaller the affected area. However, our ability to define and characterise the mechanism and rate of movement of individual compounds in the subcutaneous tissues will allow us to better predict the extent of extravasation injuries.

## 10. Discussion

The consequences of iatrogenic injuries such as those from extravasation are potentially limb-threatening and have severe ongoing consequences for the patient. Prevention, as always, is better than cure, but despite our aim to eliminate errors such as these from our hospitals, the data on incidence suggests that an approach to management needs to be clarified. There are many factors influencing the pathophysiology of the condition including those that the patient carries and those of the offending infusate. In-depth knowledge of these factors allows a tailored approach to treatment.

The initial management of extravasation injuries as outlined in Figure 1 reflects current practice in the field and provides a foundation on which to add more invasive treatments. A knowledge of the causative agent is crucial, as is an awareness of the potential for the introduction of an antidote drug. As with any iatrogenic injury, communication with the patients and their relatives is the key for maintaining trust. A firm grasp of the options for management and the current evidence for such choices alongside an appreciation of the potential progression of the injury and the prognosis for the patient aids this process.

In adults, early first aid and inclusion of the plastic surgery team for specialist advice of benefit. The type of offending agent, volume extravasated, and various patient factors influence the type of treatment that is required. This intricate mix of factors makes it difficult to accurately predict the progression of the injury and therefore the most appropriate treatment. It is the senior authors' approach to perform early surgery in the presence of skin ulcers, full-thickness skin necrosis and persistent pain. Regular review is advised until healing is achieved. This approach then allows for reconstructive surgery to follow, allowing repair of the defect and restoration of function.

## 11. Conclusion

Extravasation injury is very dangerous. It increases morbidity, causes delayed treatment of the primary disease, and has long-term sequelae. Prevention is better than cure, but where extravasation injury does occur in adults, the authors' preferred method is one of theearly surgical interventions with regular followup for the consideration of reconstructive surgery.

## Figures and Tables

**Table 1:** Medications that may increase the risk of extravasation.

Medication	Risk
Anticoagulants Antifibrinolytics Antiplatelets	May exacerbate extravasation or cause a compartmental injury by increasing local bleeding
Vasodilators	May increase local blood flow and enlarge the area of injury
Hormone Therapy	Vasodilating properties
Steroids	Vasodilating properties
Diuretics	May increase local blood flow
Antihistamines	May constrict capillaries and arterioles, resulting in ischaemic injury
Analgesics	Reduced pain sensation may cause less reporting of extravasations
IV antibiotics	Repeated venous insult may thrombose vessels

**Table 2:** Classification of chemotheraputic agents into irritants (bold), vesicants (italic) and both irritant and vesicant (normal).

**Alkylating agents **	**Cyclophosphamide **
	**Ifosfamide **
**Antimetabolites**	**Gemcitabine **
**Platinum compounds **	**Carboplatin **
**Topoisomerase inhibitors **	**Irinotecan **
	**Topotecan **

Alkylating agents	Melphalan
Antimetabolites	5-Fluorouracil
Taxanes	Docetaxel
	Paclitaxel
Other mitotic inhibitors	Bleomycin
	Etoposide

*Alkylating agents *	*Dacarbazine *
*Anthracyclines *	*Daunorubicin *
	*Doxorubicin (plus liposomal doxorubicin) *
	*Idarubicin *
*Platinum compounds *	*Cisplatin *
*Vinca alkaloids *	*Vinblastine *
	*Vincristine *
	*Vinorelbine *
*Other mitotic inhibitors *	*Dactinomycin *
	*Mitomycin *
